# The Western Ontario Meniscal Evaluation Tool Translated Into Italian Is a Reliable, Precise, and Responsive Patient-Reported Outcome Measure for Arthroscopic Meniscal Surgery

**DOI:** 10.1016/j.asmr.2025.101115

**Published:** 2025-03-13

**Authors:** Michelangelo Palco, Gabriele Giuca, Giorgio Gasparini, Roberto Simonetta, Danilo Leonetti, Filippo Familiari

**Affiliations:** a Department of Orthopaedic and Trauma Surgery, Casa di Cura Caminiti, Villa San Giovanni, Italy; b Section of Orthopaedics and Traumatology, Department of Biomedical Sciences and Morphological and Functional Images, University of Messina, Messina, Italy; c Department of Orthopaedic and Trauma Surgery and Research Center on Musculoskeletal Health, Magna Graecia University, Catanzaro, Italy

## Abstract

**Purpose:**

To translate and culturally adapt the Western Ontario Meniscal Evaluation Tool (WOMET) into Italian to examine its reliability, measurement precision, and responsiveness in patients undergoing arthroscopic meniscal surgery.

**Methods:**

Patients with magnetic resonance imaging–confirmed meniscal injuries completed the Italian WOMET at baseline and again at 3 and 6 months postoperatively. The translation followed established guidelines for cross-cultural adaptation, including forward-backward translation and cognitive debriefing. Test-retest reliability was assessed using the intraclass correlation coefficient, and measurement precision was evaluated by calculating the standard error of measurement and the minimal detectable change. Responsiveness was measured via the standardized response mean. The Knee Injury and Osteoarthritis Outcome Score 4 questionnaire was administered for comparison.

**Results:**

A total of 97 patients (mean age, 38 years; age range, 22-58 years) were included. The Italian WOMET showed excellent test-retest reliability (intraclass correlation coefficient, 0.87). The standard error of measurement was 109.68 points, and the minimal detectable change was 307 points, indicating a high level of precision for detecting true clinical changes. The standardized response means were 1.94 at 3 months and 2.44 at 6 months, indicating strong responsiveness. A high correlation (*r* = 0.85, *P* < .001) with the Knee Injury and Osteoarthritis Outcome Score 4 supported concurrent validity.

**Conclusions:**

The Italian WOMET is a reliable, precise, and highly responsive patient-reported outcome measure for assessing health-related quality of life in patients undergoing arthroscopic meniscal surgery.

**Clinical Relevance:**

Given the increasing prevalence of meniscal injuries in Italy and the need for culturally relevant diagnostic tools, the validation of the WOMET in Italian is important for patients, health care providers, and scientists.

Meniscal injuries are a common source of knee pain and functional limitations, particularly among athletes and active individuals. When left untreated, they may progress to osteoarthritis, underscoring the importance of accurate diagnosis and effective management.^1^, ^2^, ^3^, ^4^, ^5^ In recent years, the understanding of knee pathologies has expanded substantially, particularly with the use of innovative treatment approaches and surgical techniques. For example, the use of all-suture anchors in meniscal repair has shown promising outcomes in restoring knee function.^6^ Additionally, the classification systems for meniscal ramp lesions have become more refined, aiding in the surgical decision-making process.^6^, ^7^, ^8^ These advancements underscore the need for validated tools, such as the Western Ontario Meniscal Evaluation Tool (WOMET), to assess post-treatment quality of life. Patient-reported outcome measures (PROMs) have become essential in assessing the success of interventions aimed at addressing knee injuries, particularly in relation to health-related quality of life (HRQoL). PROMs such as the Knee Injury and Osteoarthritis Outcome Score (KOOS)^9^ and the International Knee Documentation Committee subjective knee form^10^ have long been used to evaluate knee function, but they lack specificity when assessing meniscal pathologies. Generalized tools often fail to capture the nuanced physical and emotional impact of meniscal injuries, as well as their effect on patients’ daily functioning and sports activities.

The WOMET^11^ is a meniscus-specific PROM that focuses on symptoms such as pain, mechanical dysfunction, and swelling, as well as the broader emotional and functional consequences of meniscal injuries in patients’ daily activities.^12^ The WOMET has been validated across multiple languages, including German,^13^ Dutch,^14^ and Turkish,^15^ all showing strong psychometric properties in terms of reliability, validity, and responsiveness. However, a validated version in Italian is not available. Given Italy’s distinct health care environment, which includes both public and private health care systems, there is a pressing need for PROMs that are not only linguistically accurate but also culturally adapted to the Italian context.

This study aimed to translate and culturally adapt the WOMET into Italian to examine its reliability, measurement precision, and responsiveness in patients undergoing arthroscopic meniscal surgery. We hypothesized that the Italian WOMET would be reliable, precise, and responsive in patients undergoing arthroscopic meniscal surgery. Additionally, the study aimed to compare the Italian WOMET’s performance with the Knee Injury and Osteoarthritis Outcome Score 4 (KOOS4), a more general knee function measure, to determine its broader clinical utility.

## Methods

### Study Design

This study followed the internationally accepted guidelines for the cross-cultural adaptation and validation of PROMs.^16^ Cross-cultural adaptation is essential to ensure that the translated version of a PROM is not only linguistically accurate but also culturally relevant to the population it is intended to assess. Ethical approval was obtained from the Territorial Ethics Committee of Calabria (institutional review board protocol No. 24/2025). Written informed consent was obtained from all participants in accordance with the Declaration of Helsinki. The online platform used for data collection (https://survey.zohopublic.eu/zs/KxBuTg) helped gather responses systematically. Data collection took place between January and June 2024, ensuring a structured and comprehensive assessment of patient-reported outcomes. This approach ensured comprehensive and structured feedback from the patients involved. The adaptation process involved several key stages, including forward translation, backward translation, and cognitive debriefing with patients to ensure the comprehensibility and relevance of the translated version. Patients completed the WOMET and KOOS4 at baseline (preoperatively) and at a follow-up interval of 6 weeks postoperatively, allowing for the evaluation of changes over time. The psychometric properties of the Italian WOMET were then evaluated through rigorous statistical testing, including assessments of reliability, measurement precision, and responsiveness.

### Translation Process

The translation of the WOMET into Italian was conducted following a standardized process. Two independent Italian-speaking translators, one with a medical background and one without, first translated the original English-language version into Italian. This approach ensured that both clinical terminology and everyday language were appropriately represented. These translations were synthesized into a single version, which was then back-translated into English by 2 native English-language speakers fluent in Italian but unfamiliar with the original WOMET. Any discrepancies were reviewed, and minimal adjustments were made to ensure semantic equivalence. No major revisions were deemed necessary.

### Subjects

A total of 97 patients (mean age, 38 years; age range, 22-58 years) with magnetic resonance imaging–confirmed meniscal injuries were recruited from the Casa di Cura Caminiti Orthopedic Department, Villa San Giovanni, Italy, an accredited hospital. This setting represents a typical orthopaedic facility within the Italian health care system, which includes both public and private institutions. All patients were scheduled for arthroscopic meniscal surgery, which is considered the gold standard for meniscal repair in cases involving mechanical symptoms or persistent pain. The exclusion criteria included patients with severe ligamentous injuries, such as anterior or posterior cruciate ligament tears; advanced osteoarthritis; or other knee conditions that could confound the results. The sample size of 97 patients was determined based on recommended guidelines for the validation of PROMs, which suggest a minimum of 50 patients for reliability testing and 100 patients for responsiveness testing.^17^

### Outcome Measures

The primary outcome measure was the Italian version of the WOMET, which assesses 3 main domains: (1) physical symptoms (pain, swelling, and mechanical symptoms), (2) emotional well-being (including anxiety and mood), and (3) impact on daily activities (Appendix Fig 1). These domains provide a comprehensive assessment of the impact of meniscal injuries on patients’ lives, particularly in the postoperative recovery period. For comparison, the KOOS4 was also administered to assess general knee function. The KOOS4 includes 4 of the 5 KOOS subscales: pain, symptoms, activities of daily living, and quality of life. However, the KOOS4 lacks the meniscus-specific metrics provided by the WOMET.

### Statistical Analysis

All statistical analyses were performed using IBM SPSS Statistics for Windows, version 29.0.2.0 (IBM, Armonk, NY). The level of significance was set at *P* < .05.

##### Reliability

Reliability, particularly test-retest reliability, is critical in determining whether a PROM consistently measures the same construct over time in the absence of true clinical change. To assess test-retest reliability, the intraclass correlation coefficient (ICC) was calculated. ICC values above 0.75 indicate excellent reliability, with values approaching 1.0 representing near-perfect agreement between test and retest scores.^18^

##### Measurement Precision

The standard error of measurement (SEM) quantified random measurement error. The minimal detectable change (MDC) at the 95% confidence level was calculated as 1.96 × SEM × √2. This reflects the smallest change not due to chance.

##### Responsiveness

Responsiveness refers to the ability of a PROM to detect changes in a patient’s condition over time, particularly in response to interventions such as surgery or rehabilitation. To assess the responsiveness of the Italian WOMET, the standardized response mean (SRM) was calculated. An SRM greater than 0.8 is considered to indicate a large effect, meaning that the PROM is highly responsive to changes in the patient’s condition. For comparison, the responsiveness of the KOOS4 was also evaluated.

##### Validity

Pearson correlation (*r*) assessed the relationship between WOMET and KOOS4 scores. Bland-Altman plots were constructed to evaluate the agreement between test and retest scores and to identify any systematic bias or outliers.

## Results

### Translation and Cultural Adaptation

The translation and cultural adaptation of the WOMET into Italian proceeded smoothly, with no major linguistic or cultural discrepancies encountered during the forward-backward translation process. No major discrepancies were found during this process, and participants in cognitive debriefing confirmed item clarity and relevance.

### Reliability

The Italian WOMET showed excellent test-retest reliability (ICC, 0.87). Bland-Altman plots showed minimal bias between measurements, with tight limits of agreement (Table 1).

**Table 1:** Clear and Concise Overview of Key Metrics Such as ICC, SEM, MDC, and Effect Sizes for WOMET and KOOS4

Psychometric Property	Description	Value
Reliability		
ICC	Measures reliability or agreement between test and retest scores	0.87 (excellent)
Measurement precision		
SEM	Reflects precision of individual scores on WOMET	109.68 points
MDC	Indicates smallest change that exceeds measurement error, indicating real change in patient status	307 points
Responsiveness (SRM)	SRM > 0.8 considered large effect size, indicating high responsiveness	
3 mo after surgery		1.94 (large effect)
6 mo after surgery		2.44 (large effect)
Validity		
Correlation with KOOS4: Pearson *r*	Pearson *r* value of 0.85 denotes strong positive correlation between WOMET and KOOS4 scores	0.85 (*P* < .0001)

ICC, intraclass correlation coefficient; KOOS4, Knee Injury and Osteoarthritis Outcome Score 4; MDC; minimal detectable change; SEM, standard error of measurement; SRM, standardized response mean; WOMET, Western Ontario Meniscal Evaluation Tool.

**Fig 1:**
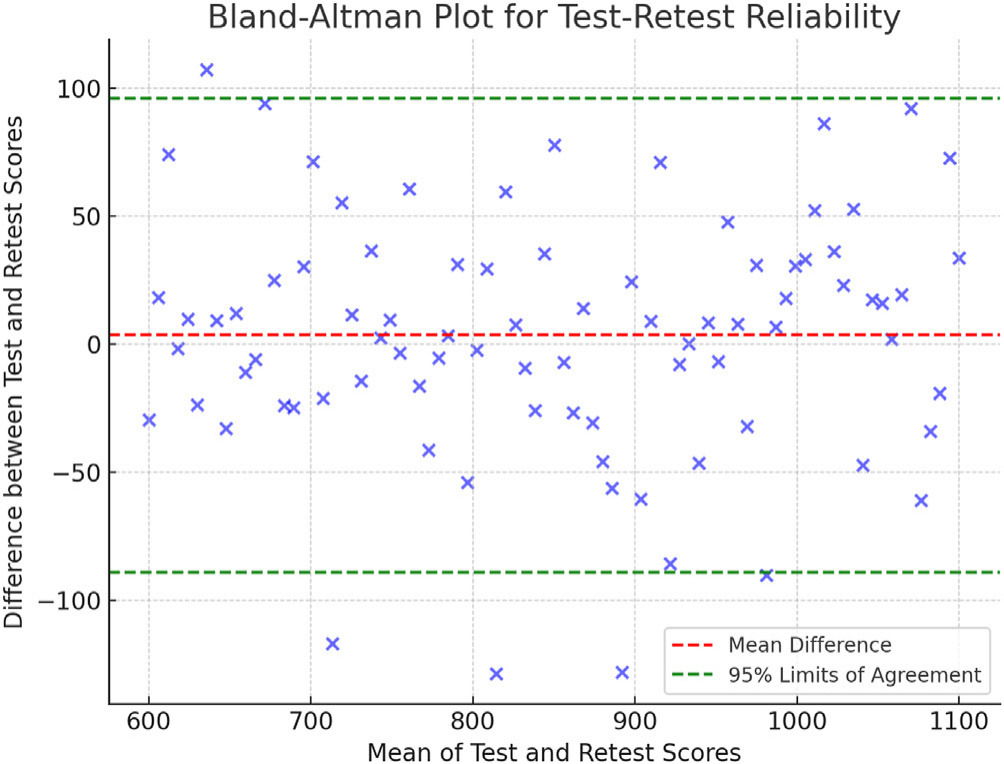
A Bland-Altman plot shows excellent agreement between test and retest scores for the Italian Western Ontario Meniscal Evaluation Tool, confirming its test-retest reliability. Tight clustering of values around the zero line suggests minimal measurement bias.

This level of reliability is consistent with previous validation studies of the WOMET, including those conducted in Germany^13^ and Denmark,^15^ in which ICC values ranged from 0.88 to 0.90. To further assess the agreement between test and retest scores, Bland-Altman plots were used, showing no significant bias between the 2 time points. The limits of agreement showed tight clustering of values, suggesting the tool’s stability over time and its capacity to consistently measure HRQoL in patients with meniscal injuries (Fig 1).

### Measurement Precision

**Fig 2:**
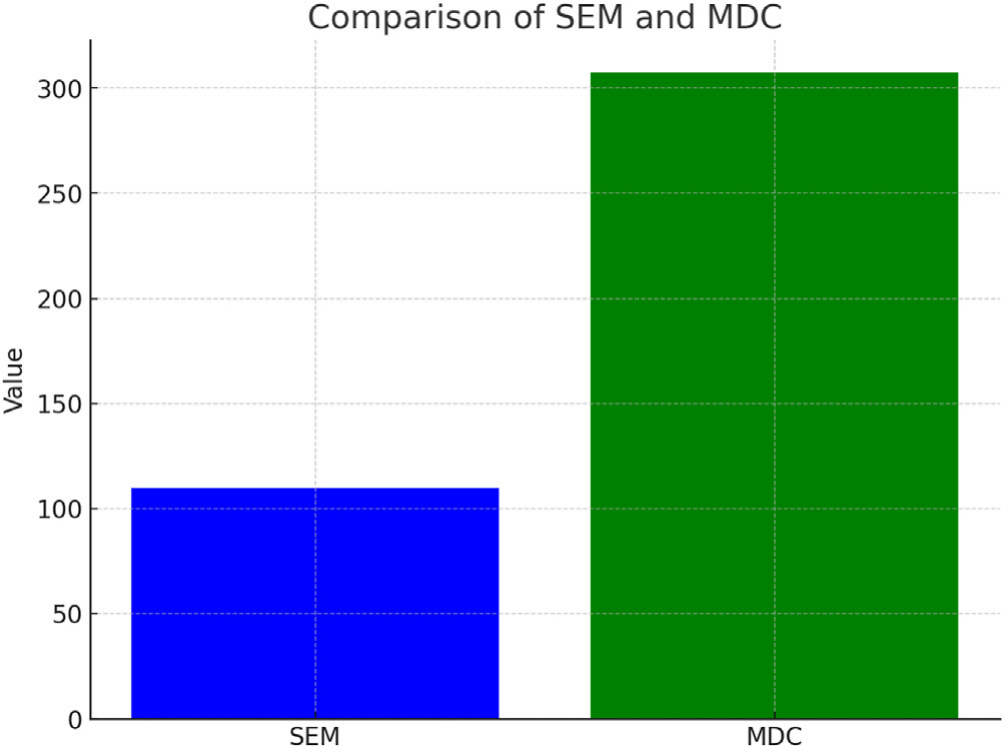
A bar chart illustrates the precision of the tool, with the standard error of measurement (SEM) at 3.04 points and the minimal detectable change (MDC) at 8.43 points, demonstrating the ability of the Western Ontario Meniscal Evaluation Tool (WOMET) to detect clinically meaningful changes in patients’ conditions.

Measurement precision was evaluated using the SEM and the MDC. The SEM was calculated to be 109.68 points, whereas the MDC was determined to be 307 points. Any improvement exceeding 307 points can be interpreted as a true clinical change (Fig 2).

### Responsiveness

**Fig 3:**
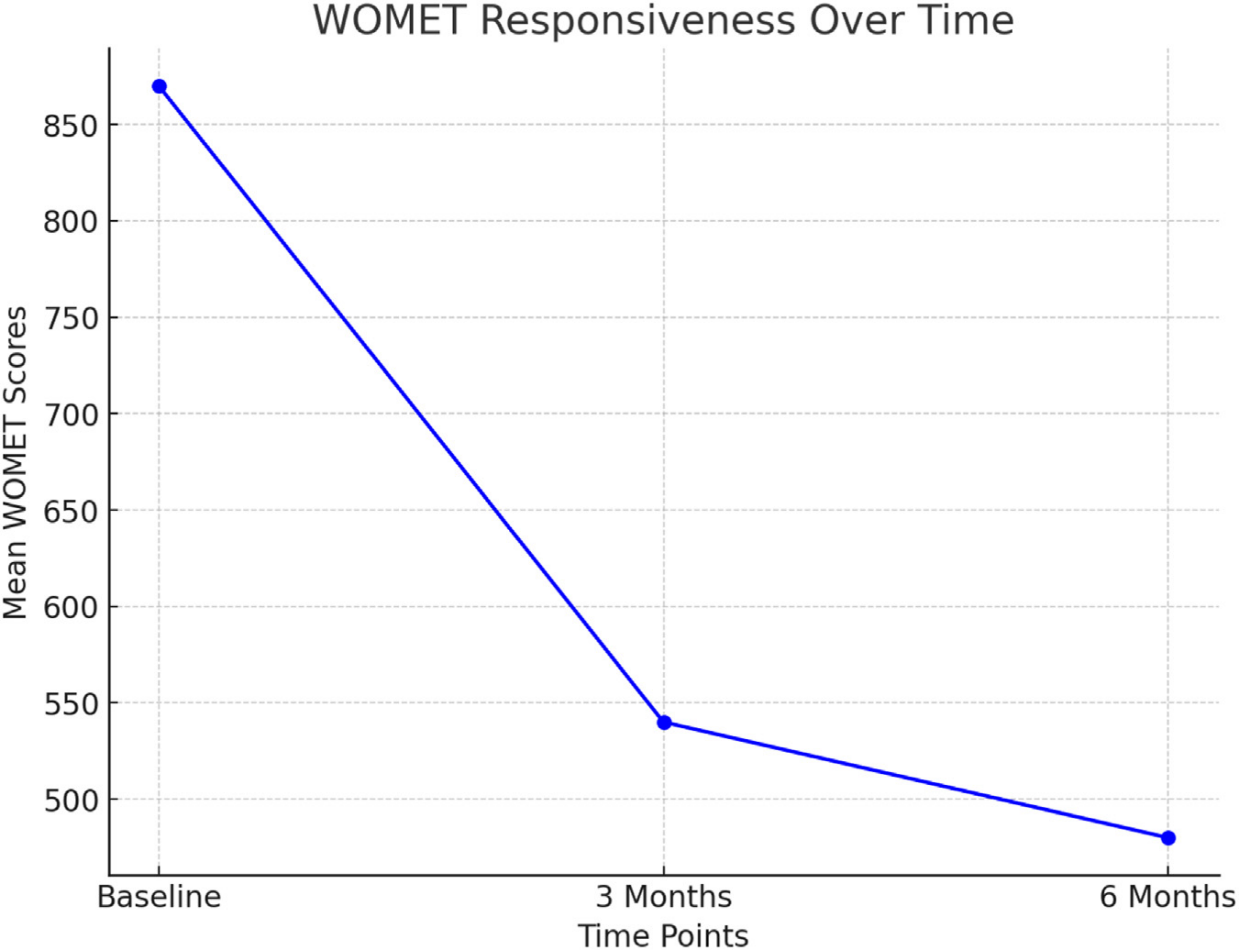
A line graph presents changes in Western Ontario Meniscal Evaluation Tool (WOMET) scores from baseline to 6 months after surgery, showing a significant improvement, which highlights the tool’s strong responsiveness.

Responsiveness was assessed by calculating the SRM for changes in WOMET scores from baseline to 6 months after surgery. The Italian WOMET showed strong responsiveness, with SRMs of 1.94 at 3 months and 2.44 at 6 months, indicating significant clinical improvement over time. Comparatively, the KOOS4 exhibited a slightly lower SRM of 1.10. These findings highlight the Italian WOMET’s sensitivity to postoperative changes in meniscal injury recovery (Fig 3).

### Correlation With KOOS4

**Fig 4:**
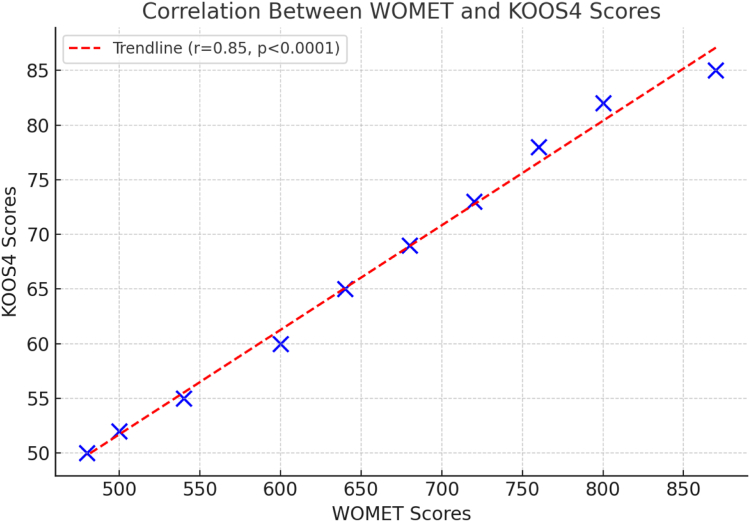
A plot shows the correlation between Western Ontario Meniscal Evaluation Tool (WOMET) scores and Knee Injury and Osteoarthritis Outcome Score 4 (KOOS4) values (*r* = 0.85, *P* < .001), further validating the clinical utility of the Italian WOMET.

The strong correlation with the KOOS4 (*r* = 0.85, *P* < .001) supports the concurrent validity of the Italian WOMET. Whereas the KOOS4 captures general knee function, the WOMET provides a more focused assessment of meniscal pathologies (Fig 4).

## Discussion

In this study, we validated the Italian version of the WOMET as a reliable and responsive tool for evaluating HRQoL in patients undergoing meniscal surgery. The tool’s high ICC (0.87), excellent precision (SEM, 109.68 points), and strong responsiveness (SRM, 2.44) shows its utility in tracking postoperative recovery. These findings align with previous validations in other languages, such as German (ICC, 0.90),^13^ Turkish (ICC, 0.88),^15^ Chinese (ICC, 0.93),^19^ and Danish (ICC, 0.88).^20^ The consistency across different cultural contexts shows the robustness of the WOMET as a reliable tool for assessing HRQoL in meniscal injury patients, regardless of language or cultural differences.

Our study also revealed that the Italian WOMET is highly responsive to clinical improvements, as reflected by its SRM of 2.44. This result is consistent with responsiveness values reported in other translated versions, such as the Dutch and Danish WOMET,^14^^,^^20^ confirming that the WOMET is not only reliable but also sensitive in detecting meaningful changes in patients’ conditions, particularly after surgical interventions. This responsiveness makes it a valuable tool for clinicians who need to monitor postoperative recovery and adapt rehabilitation protocols based on patient progress.

The strong correlation between the WOMET and KOOS4 (*r* = 0.85) confirms that the Italian WOMET captures not only meniscus-specific outcomes but also general knee function, making it versatile for clinical use. Its ability to capture detailed aspects of meniscal pathology, such as mechanical symptoms and their emotional consequences, distinguishes it from other knee PROMs such as the International Knee Documentation Committee and Tegner-Lysholm scales, which are broader in scope.

### Clinical Implications

Given the increasing prevalence of meniscal injuries in Italy and the need for culturally relevant diagnostic tools, the validation of the WOMET in Italian is particularly timely. Our findings are consistent with those of recent comparative studies on knee osteoarthritis treatments, such as the combined use of platelet-rich plasma and hyaluronic acid, which have shown improvements in pain management and functional outcomes.^21^^,^^22^ Moreover, the benefits of using anterior versus posterior referencing techniques in knee prosthesis surgery^21^^,^^23^ support the importance of precise surgical techniques to optimize postoperative recovery. Finally, the growing body of literature on the classification of complex meniscal lesions further validates our approach to evaluating meniscal injuries and their outcomes using tools such as the WOMET.^21^^,^^24^ The validation of the Italian WOMET not only provides a valuable tool for clinicians and researchers but also opens new avenues for future studies on meniscal injury outcomes. Given its strong psychometric properties, the tool can be used to evaluate the long-term outcomes of various meniscal treatments, including surgical and nonsurgical interventions. Moreover, integrating the WOMET with other knee-specific PROMs in multicenter trials may offer a more comprehensive assessment framework for knee injuries, particularly in cases involving multiple concurrent pathologies or complex interventions.

Although this study provides strong evidence of the tool’s reliability and responsiveness, future research should aim to validate the Italian WOMET in larger, multicenter studies to ensure its applicability across diverse health care settings in Italy. Such studies could also explore integrating the WOMET with other PROMs to create a more comprehensive assessment framework for knee injuries, especially in cases involving complex pathologies or concurrent procedures such as ligament reconstruction.

The Italian version of the WOMET offers clinicians a reliable, precise, and responsive tool for assessing the impact of meniscal injuries on patients’ quality of life. Its strong psychometric properties make it indispensable for clinical practice and research, laying a foundation for future studies aimed at improving the management and treatment of meniscal injuries in Italy.

### Limitations

Several limitations should be noted. First, the study was conducted at a single center, which may limit the generalizability of the results to broader clinical settings. Second, patients with concurrent ligamentous injuries, such as anterior or posterior cruciate ligament tears, were excluded, potentially omitting individuals with more complex knee injuries who might benefit from the WOMET. Additionally, although the translation process followed rigorous guidelines, certain cultural nuances may still influence the subjective interpretation of specific items, warranting further validation in a more diverse patient population.

## Conclusions

The Italian WOMET is a reliable, precise, and highly responsive PROM for assessing HRQoL in patients undergoing arthroscopic meniscal surgery.

## Disclosures

All authors (M.P., G.G., G.G., R.S., D.L., F.F.) declare that they have no known competing financial interests or personal relationships that could have appeared to influence the work reported in this paper.
