# Supplementary material for: The Western Ontario Meniscal Evaluation Tool Translated Into Italian Is a Reliable, Precise, and Responsive Patient-Reported Outcome Measure for Arthroscopic Meniscal Surgery
Source: Arthrosc Sports Med Rehabil. 2025 Mar 13;7(3):101115. doi: 10.1016/j.asmr.2025.101115 (PMC12276577; doi:10.1016/j.asmr.2025.101115)
Supplement: Supplementary Data [file mmc1.pdf]

Zoho Survey

WESTERN ONTARIO MENISCAL EVALUATION TOOL (WOMET)

ISTRUZIONI PER I PAZIENTI

Nelle Sezioni A, B, C, e D le verrà richiesto di rispondere alle domande posizionando il pallino sulla linea orizzontale.

Viene richiesto di indicare sul questionario quanto si è manifestato il sintomo correlato alla patologia del ginocchio nell'ultima settimana.

Se per qualche motivo non si comprende una domanda, si prega di far riferimento alle spiegazioni che si trovano di seguito.

Spiegazione del significato delle domande presenti nel questionario WOMET

Sintomi Fisici

1. Riferita alla sensazione che il ginocchio non regga o sia instabile.

2. Riferita al dolore o indolenzimento che si prova dopo aver svolto attività sportive, lavorative o attività domestiche.

3. Riferita al non essere in grado di flettere o estendere completamente il ginocchio.

4. Riferita alla sensazione di intorpidimento nell'area del ginocchio o attorno alla cicatrice.

5. Riferita alla sensazione di difficoltà nell'eseguire un movimento del ginocchio. Questo non si riferisce alla perdita di movimento.

6. Riferita ad una mancanza di forza o debolezza del ginocchio nel compiere un'azione.

7. Riferita a qualsiasi aumento di dimensione del ginocchio per il gonfiore.

8. Riferita al dolore che si prova quando si sta in piedi o si svolgono attività che richiedono l'appoggio del pieno carico sul ginocchio.

9. Riferita a qualsiasi rumore provenga dal ginocchio quando si piega o si cammina ecc.

Sport/Attività Ricreative/Lavoro/Stile di Vita

10. Riferita alla preoccupazione di una nuova lesione del menisco nello svolgere la stessa pratica sportiva o lavorativa precedente all'infortunio.

11. Riferita alla necessità di poter dedicare meno tempo ad attività che venivano svolte prima dell'infortunio o di doverle sospendere per scarsa resistenza o dolore, ecc. al ginocchio.

12. Riferita a qualsiasi difficoltà nello svolgere gesti richiesti nel lavoro, nello sport e nelle attività ricreative o domestiche.

13. Riferita al non potersi accovacciare a causa del dolore o all'incapacità di flettere completamente il ginocchio.

Sensazioni

14. Riferita alla continua apprensione per il proprio ginocchio o alla necessità di prenderlo in considerazione prima di intraprendere qualsiasi attività.

15. Riferita alla preoccupazione che le condizioni del ginocchio possano peggiorare nell'immediato o a lungo termine con l'invecchiamento.

16. Riferita alla frustrazione o allo sconforto derivanti dall'incapacità di svolgere attività prima usuali o che si ha intenzione di svolgere ma non fattibili a causa del ginocchio

- [ ] Ho preso visione

Inizia sondaggio

Tecnologia Zoho Survey (http://zoho.com/survey)
Crea gratuitamente un numero illimitato di sondaggi online

Se trovi dati riservati in questo sondaggio che ritieni potrebbero creare danni, invia una segnalazione qui (https://www.zoho.eu/report-abuse/)

Zoho Survey

WESTERN ONTARIO MENISCAL EVALUATION TOOL (WOMET)

Sezione A

Sintomi Fisici

* Quanto sei stato infastidito da sensazione di cedimento o insicurezza del ginocchio?

0 100

Per niente Estremamente infastidito

Si prega di inserire quanto si è manifestato il sintomo nell'ultima settimana

0 / 5000

Successivo

Non condividere nessuna informazione relativa alle password in questo sondaggio

Tecnologia Zoho Survey (http://zoho.com/survey)

Crea gratuitamente un numero illimitato di sondaggi online

Se trovi dati riservati in questo sondaggio che ritieni potrebbero creare danni, invia una segnalazione qui (https://www.zoho.eu/report-abuse/)

Zoho Survey

WESTERN ONTARIO MENISCAL EVALUATION TOOL (WOMET)

Sezione A

Sintomi Fisici

* Quanto sei stato infastidito da dolore o indolenzimento del ginocchio dopo le attività?

0 100

Per niente

Estremamente infastidito

Si prega di inserire quanto si è manifestato il sintomo nell'ultima settimana

0 / 5000

Precedente Successivo

Non condividere nessuna informazione relativa alle password in questo sondaggio

Tecnologia Zoho Survey (http://zoho.com/survey)

Crea gratuitamente un numero illimitato di sondaggi online

Se trovi dati riservati in questo sondaggio che ritieni potrebbero creare danni, invia una segnalazione qui (https://www.zoho.eu/report-abuse/)

Zoho Survey

WESTERN ONTARIO MENISCAL EVALUATION TOOL (WOMET)

Sezione A

Sintomi Fisici

* Quanto sei stato infastidito da perdita di movimento del ginocchio?

0 100

Per niente — Estremamente infastidito

Si prega di inserire quanto si è manifestato il sintomo nell’ultima settimana

0 / 5000

Precedente Successivo

Non condividere nessuna informazione relativa alle password in questo sondaggio

Tecnologia Zoho Survey (http://zoho.com/survey)

Crea gratuitamente un numero illimitato di sondaggi online

Se trovi dati riservati in questo sondaggio che ritieni potrebbero creare danni, invia una segnalazione qui (https://www.zoho.eu/report-abuse/)

Zoho Survey

WESTERN ONTARIO MENISCAL EVALUATION TOOL (WOMET)

Sezione A

Sintomi Fisici

*Quanto sei stato infastidito da intorpidimento del ginocchio o delle regioni circostanti?

0 100

Per niente

Estremamente infastidito

Si prega di inserire quanto si è manifestato il sintomo nell’ultima settimana

0 / 5000

Precedente Successivo

Non condividere nessuna informazione relativa alle password in questo sondaggio

Tecnologia Zoho Survey (http://zoho.com/survey)

Crea gratuitamente un numero illimitato di sondaggi online

Se trovi dati riservati in questo sondaggio che ritieni potrebbero creare danni, invia una segnalazione qui (https://www.zoho.eu/report-abuse/)

WESTERN ONTARIO MENISCAL EVALUATION TOOL (WOMET)

Sezione A

Sintomi Fisici

*

Quanto sei stato infastidito da rigidità del ginocchio dopo il risveglio al mattino o dopo essere stato seduto per lungo tempo?

0 100

Per niente

Estremamente infastidito

Si prega di inserire quanto si è manifestato il sintomo nell'ultima settimana

0 / 5000

Precedente Successivo

Non condividere nessuna informazione relativa alle password in questo sondaggio

Tecnologia Zoho Survey (http://zoho.com/survey)

Crea gratuitamente un numero illimitato di sondaggi online

Se trovi dati riservati in questo sondaggio che ritieni potrebbero creare danni, invia una segnalazione qui (https://www.zoho.eu/report-abuse/)

Zoho Survey

WESTERN ONTARIO MENISCAL EVALUATION TOOL (WOMET)

Sezione A

Sintomi Fisici

* Quanto sei stato infastidito da perdita di forza al ginocchio?

0 100

Per niente

Estremamente infastidito

Si prega di inserire quanto si è manifestato il sintomo nell'ultima settimana

0 / 5000

Precedente Successivo

Non condividere nessuna informazione relativa alle password in questo sondaggio

Tecnologia Zoho Survey (http://zoho.com/survey)
Crea gratuitamente un numero illimitato di sondaggi online

Se trovi dati riservati in questo sondaggio che ritieni potrebbero creare danni, invia una segnalazione qui (https://www.zoho.eu/report-abuse/)

Zoho Survey

WESTERN ONTARIO MENISCAL EVALUATION TOOL (WOMET)

Sezione A

Sintomi Fisici

* Quanto sei stato infastidito da gonfiore al ginocchio?

0 100

Per niente Estremamente infastidito

Si prega di inserire quanto si è manifestato il sintomo nell’ultima settimana

0 / 5000

Precedente Successivo

Non condividere nessuna informazione relativa alle password in questo sondaggio

Tecnologia Zoho Survey (http://zoho.com/survey)

Crea gratuitamente un numero illimitato di sondaggi online

Se trovi dati riservati in questo sondaggio che ritieni potrebbero creare danni, invia una segnalazione qui (https://www.zoho.eu/report-abuse/)

Zoho Survey

WESTERN ONTARIO MENISCAL EVALUATION TOOL (WOMET)

Sezione A

Sintomi Fisici

* Quanto sei stato infastidito da dolore trafittivo al ginocchio dopo averlo sottoposto a pieno carico per un periodo di tempo?

0 100

Per niente

Estremamente infastidito

Si prega di inserire quanto si è manifestato il sintomo nell'ultima settimana

0 / 5000

Precedente Successivo

Non condividere nessuna informazione relativa alle password in questo sondaggio

Tecnologia Zoho Survey (http://zoho.com/survey)

Crea gratuitamente un numero illimitato di sondaggi online

Se trovi dati riservati in questo sondaggio che ritieni potrebbero creare danni, invia una segnalazione qui (https://www.zoho.eu/report-abuse/)

Zoho Survey

WESTERN ONTARIO MENISCAL EVALUATION TOOL (WOMET)

Sezione A

Sintomi Fisici

* Quanto sei stato infastidito da crepitii, scricchiolii o schiocchi del ginocchio?

0 100

Per niente

Estremamente infastidito

Si prega di inserire quanto si è manifestato il sintomo nell'ultima settimana

0 / 5000

Precedente Successivo

Non condividere nessuna informazione relativa alle password in questo sondaggio

Tecnologia Zoho Survey (http://zoho.com/survey)

Crea gratuitamente un numero illimitato di sondaggi online

Se trovi dati riservati in questo sondaggio che ritieni potrebbero creare danni, invia una segnalazione qui (https://www.zoho.eu/report-abuse/)

Zoho Survey

WESTERN ONTARIO MENISCAL EVALUATION TOOL (WOMET)

Sezione B

Sport/Attività ricreative/Lavoro/Stile di Vita

* Quanto sei preoccupato da un re-infortunio del ginocchio in seguito alla ripresa dell'attività lavorativa o sportiva?

0 100

Per niente

Estremamente preoccupato

Precedente Successivo

Non condividere nessuna informazione relativa alle password in questo sondaggio

Tecnologia Zoho Survey (http://zoho.com/survey)
Crea gratuitamente un numero illimitato di sondaggi online

Se trovi dati riservati in questo sondaggio che ritieni potrebbero creare danni, invia una segnalazione qui (https://www.zoho.eu/report-abuse/)

Zoho Survey

WESTERN ONTARIO MENISCAL EVALUATION TOOL (WOMET)

Sezione B

Sport/Attività ricreative/Lavoro/Stile di Vita

* Quanto ha influito il ginocchio sulla quantità di tempo che dedichi alle attività che svolgevi anche prima dell'infortunio?

0 100

Per niente Estremamente

Precedente Successivo

Non condividere nessuna informazione relativa alle password in questo sondaggio

Tecnologia Zoho Survey (http://zoho.com/survey)
Crea gratuitamente un numero illimitato di sondaggi online

Se trovi dati riservati in questo sondaggio che ritieni potrebbero creare danni, invia una segnalazione qui (https://www.zoho.eu/report-abuse/)

Zoho Survey

WESTERN ONTARIO MENISCAL EVALUATION TOOL (WOMET)

Sezione B

Sport/Attività ricreative/Lavoro/Stile di Vita

*Quanto ha influito il ginocchio sulla capacità di eseguire specifici gesti richiesti dal tuo sport o lavoro?

(Se coinvolti entrambi, si consideri l'ambito che è stato maggiormente influenzato).

0 100

Per niente Estremamente

Precedente Successivo

Non condividere nessuna informazione relativa alle password in questo sondaggio

Tecnologia Zoho Survey (http://zoho.com/survey)

Crea gratuitamente un numero illimitato di sondaggi online

Se trovi dati riservati in questo sondaggio che ritieni potrebbero creare danni, invia una segnalazione qui (https://www.zoho.eu/report-abuse/)

WESTERN ONTARIO MENISCAL EVALUATION TOOL (WOMET)

Sezione B

Sport/Attività ricreative/Lavoro/Stile di Vita

*

Quanto risulta problematico l'accovacciamento?

0 100

Per niente Estremamente

Precedente Successivo

Non condividere nessuna informazione relativa alle password in questo sondaggio

Tecnologia Zoho Survey (http://zoho.com/survey)
Crea gratuitamente un numero illimitato di sondaggi online

Se trovi dati riservati in questo sondaggio che ritieni potrebbero creare danni, invia una segnalazione qui (https://www.zoho.eu/report-abuse/)

Zoho Survey

WESTERN ONTARIO MENISCAL EVALUATION TOOL (WOMET)

Sezione C

Sensazioni

*

Quanto pensi al tuo ginocchio?

0 100

Per niente Estremamente

Precedente Successivo

Non condividere nessuna informazione relativa alle password in questo sondaggio

Tecnologia Zoho Survey (http://zoho.com/survey)

Crea gratuitamente un numero illimitato di sondaggi online

Se trovi dati riservati in questo sondaggio che ritieni potrebbero creare danni, invia una segnalazione qui (https://www.zoho.eu/report-abuse/)

Zoho Survey

WESTERN ONTARIO MENISCAL EVALUATION TOOL (WOMET)

Sezione C

Sensazioni

* Quanto sei preoccupato per ciò che succederà al tuo ginocchio in futuro?

0 100

Per niente Estremamente

Precedente Successivo

Non condividere nessuna informazione relativa alle password in questo sondaggio

Tecnologia Zoho Survey (http://zoho.com/survey)
Crea gratuitamente un numero illimitato di sondaggi online

Se trovi dati riservati in questo sondaggio che ritieni potrebbero creare danni, invia una segnalazione qui (https://www.zoho.eu/report-abuse/)

Zoho Survey

WESTERN ONTARIO MENISCAL EVALUATION TOOL (WOMET)

Sezione C

Sensazioni

* Quanta frustrazione o sconforto provi a causa del tuo ginocchio?

0 100

Per niente Estrema

Precedente Invia

Non condividere nessuna informazione relativa alle password in questo sondaggio

Tecnologia Zoho Survey (http://zoho.com/survey)
Crea gratuitamente un numero illimitato di sondaggi online

Se trovi dati riservati in questo sondaggio che ritieni potrebbero creare danni, invia una segnalazione qui (https://www.zoho.eu/report-abuse/)

Hai già un account? Accedi

Grazie!

La tua risposta è stata inviata

Vuoi crearne uno?

PROVA ZOHO SURVEY
